# Bitumen Recovery from Crude Bitumen Samples from Halfaya Oilfield by Single and Composite Solvents—Process, Parameters, and Mechanism

**DOI:** 10.3390/ma12172656

**Published:** 2019-08-21

**Authors:** Yunfeng Liu, Zhengsong Qiu, Hanyi Zhong, Zhen Nie, Jia Li, Weian Huang, Xin Zhao

**Affiliations:** 1 School of Petroleum Engi neering, China University of Petroleum (East China), Qingdao 266580, China; 2 China National Petroleum Corporation (CNPC) Research Institute of Petroleum Exploration and Development, Beijing 100083, China

**Keywords:** bitumen recovery, solvent extraction, SARA analysis, composite solvent

## Abstract

Since 2007, heterogeneous, high-viscosity active bituminous formations have often occurred during the drilling process in Yadavaran oilfield (Iran), Halfaya oilfield (Iraq), and tar sands (Canada). The formation of bitumen exhibits plastic and creep properties, and its adhesion is strong, so drilling accidents are easily caused, such as adhering vibrating screen, drill pipe sticking, lost circulation, and even well abandonment. These complex problems cause huge economic losses. Solvents used to dissolve bitumen are a feasible technology to remove bitumen effectively. In order to solve this problem, we used crude bitumen samples from Halfaya oilfield to study the relation between the bitumen component and different solvents. In this study, the temperature, crude bitumen sample to solvent ratio, stirring rate, stirring time, and ultrasound time on bitumen recovery by toluene were investigated by a single factor experiment. The optimum process parameter for bitumen recovery was obtained. Toluene, n-heptane, tetrahydrofuran, cyclohexane, cyclopentane, ethyl acetate, and n-pentane were chosen as the solvents for single solvent extraction and composite solvent extraction. The bitumen recovery increased significantly with the use of a composite solvent compared to a single solvent. The composite solvent ratio was 1:1. The highest bitumen recovery was 98.9 wt% by toluene/cyclohexane composite solvent. The SARA (saturates, aromatics, resins, and asphaltenes) components of the bitumen were analyzed. The toluene showed the highest asphaltene content, while the n-alkanes showed the lowest asphaltene content. The higher the asphaltene content, the higher the bitumen recovery. The composite solvent obtained the highest asphaltene content and bitumen recovery. The viscosity of bitumen extraction by different solvents was measured. The lower the bitumen viscosity, the higher the bitumen recovery. The element analysis indicated the solvent’s ability to extract bitumen colloids with the C/H ratio. This study provides a reliable theoretical basis for the subsequent adoption of effective anti-bitumen polluted drilling fluid additives.

## 1. Introduction

**Figure 1:**
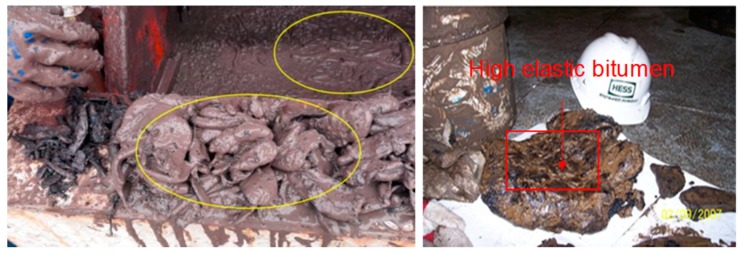
Typical formation of bitumen drilled in the oil-gas drilling process.

Since 2007, heterogeneous and active bituminous formations have been encountered in the development of Yadavaran oilfield in Iran, Halfaya oilfield in Iraq, the deep-water oilfield in the Gulf of Mexico, the tar sands in Canada, etc. [1,2,3,4,5,6]. Bituminous formations have plastic and creep properties, and the bitumen adhesion property is always strong (Figure 1). This property can easily cause several safety accidents, such as viscose vibrating screen, drilling pipe sticking, lost circulation, and even well abandonment. 

Considering the complexities in the drilling process for bituminous formations, drilling engineers often take some technical measures, including increasing the density of drilling fluids, adding diesel and emulsifiers, partially replacing the contaminated pulp, or combining chemical plugging [7].

**Figure 2:**
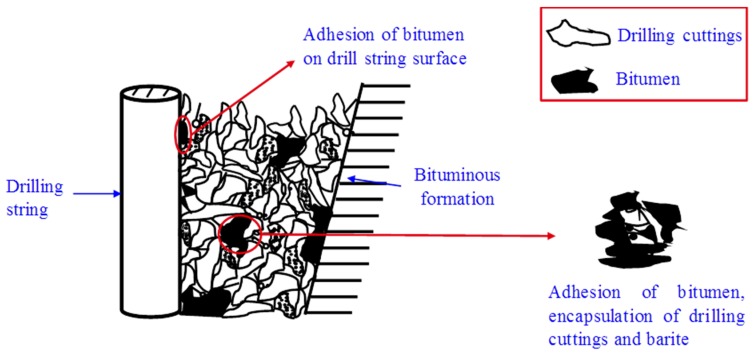
Schematic diagram of the formation of bitumen pollution and the sticking mechanism.

Through investigating the influence of drilling fluid (contaminated by the formation of bitumen) performance (Figure 2), it was shown that bitumen formation on drilling fluid caused the rheological properties of bitumen to deteriorate seriously. Moreover, the viscosity increased significantly and the energy consumption increased significantly. As was shown in Figure 2, the bitumen density varied greatly, which affected the drilling fluid circulation, and could well kick and leak occurrences. The bitumen caused the lubrication performance to deteriorate, and could even cause the drill pipe to break. The bitumen pollution made the next operation process difficult, and the harm caused is more serious than water pollution [8]. 

Therefore, it is important to find methods to remove formation bitumen. The solvent extraction of bitumen proved to be an important method to remove bitumen. Research was mainly concentrated on the oil sands separation process.

Oil sands are significant unconventional oils [9,10], and the utilization of unconventional oil is a hot research issue [11]. The methods of obtaining bitumen from oil sands consist of the hot water-based extraction (HWBE) process [12,13,14,15], the solvent extraction process [16,17], and pyrolysis [18,19,20,21]. Emulsion is usually used in the oil sands separation or enhanced oil recovery (EOR) processes [22,23,24,25]. Oil sands are categorized as oil-wet, water-wet, and neuter-wet. The HWBE process is only suitable for water-based oil sands and not suitable for oil-wet sands [26]. The HWBE process has many disadvantages, such as high energy consumption and environmental pollution. Bitumen recovery by solvent extraction shows more advantages than the HWBE process. 

Due to the differences between oil sands and crude bitumen samples from Halfaya oilfield (Iraq), the conclusions reached from oil sands solvent extraction were not suitable for crude bitumen; therefore, crude bitumen samples from Halfaya oilfield (Iraq) were chosen for the solvent extraction process and the mechanism that guides the bitumen removal process. 

Although many research studies have focused on the operational parameters of bitumen recovery, they were incomplete. Bitumen can be divided into four SARA (saturates, aromatics, resins, and asphaltenes) components, which are different in polarity, solubility, density, and molecular weight [10,27,28]. The relationship between SARA content and bitumen recovery is unclear; therefore, exploring the relationship between SARA content and bitumen recovery is important to optimizing the bitumen recovery process. 

Many researchers have focused on the mechanical properties of asphaltic materials, which constitute a significant issue for the application of bitumen in pavement design. Bazzaz put forward a procedure to characterize the nonlinear viscoelastic response of asphalt concentration at high temperatures [29]. Darabi put forward a coupled nonlinear viscoelastic (VE)- viscoplastic(VP)- hardening-relaxation(HR) model and proposed a systematic analysis procedure to study the nonlinear viscoelastic-viscoplastic with a hardening–relaxation constitutive relationship for asphalt mixtures [30]. In this paper, we focused on the viscosity of bitumen from different extraction processes, the relationship between bitumen viscosity and bitumen recovery, and the mechanical properties of bitumen. 

Previous studies have focused on a single solvent to extract bitumen from oil sands, with most research works focused on toluene’s role in bitumen recovery from oil sands. Toluene has many disadvantages; it is toxic, flammable, and causes harm to the surroundings. Therefore, finding other low-toxic solvents is a vital issue for researchers. We studied the role of different solvents on the bitumen recovery from crude bitumen samples taken from Halfaya oilfield (Iraq). The composite solvent was designed to study the effect on bitumen recovery from crude bitumen samples. 

In this study we explored the bitumen role with different solvents, in order to further solve the problem of bitumen sticking. Besides, we obtained the optimal operation parameters for bitumen recovery from crude bitumen samples by toluene extraction. In the end, we identified the bitumen recovery and SARA of single solvent extraction and composite solvent extraction.

## 2. Materials and Methods 

### 2.1. Chemicals and Samples 

Toluene, n-heptane, tetrahydrofuran, cyclohexane, cyclopentane, ethyl acetate, n-heptane, methanol, and trichloroethylene were of analytical grade and were purchased from Qingdao Baoze Technology Co. Ltd., Qingdao, China. The crude bitumen samples were from Halfaya oilfield (Iraq) (23.8 wt% sands).

### 2.2. Single Factor Experiment

The solvent extraction conditions were optimized using single factor experiments. Temperature, crude bitumen sample to solvent ratio, stirring rate, stirring time, and ultrasound time were the parameters for bitumen recovery from the crude bitumen samples.

The detailed experiment procedure is documented in Table 1 and Table 2. Table 1 shows the experimental conditions for single solvent extraction. The other factors in Table 1 are explained in detail in Table 2. First, 2.5 g of crude bitumen samples and 50 mL of toluene were weighed and put into a 200 mL beaker. The beaker was then put into a water bath at the set temperature, which was agitated in a magnetic blender. After the stirring process, the mixture was centrifuged at 7000 rpm for 15 min, and the supernatant was transferred into a flask. The solvent was removed by a rotary evaporator and the bitumen was oven-dried and then weighed. Similar experiment procedures were repeated twice. The third extraction was the final bitumen recovery. 

### 2.3. Single Solvent Extraction and Composite Solvent Extraction

The optimum technical conditions for bitumen recovery from crude bitumen samples by toluene were as follows: the temperature was 40 °C, crude bitumen sample to solvent ratio was 1:10, stirring rate was 500 rpm, stirring time was 60 min, ultrasound time was 30 min. Other solvents (n-heptane, tetrahydrofuran, cyclohexane, cyclopentane, ethyl acetate, n-pentane) were used to extract bitumen from crude bitumen samples at the optimum conditions, and the extraction process was repeated twice, as described in Section 2.2.

The composite solvents, including toluene/n-heptane, cyclohexane/cyclopentane, tetrahydrofuran/n-pentane, toluene/ethyl acetate, toluene/tetrahydrofuran, cyclohexane/ethyl acetate, and toluene/cyclohexane, were used to extract bitumen from crude bitumen samples at the optimum conditions. All of the composite solvents were mixed at a ratio of 1:1, and then the composite solvents were used to extract bitumen from crude bitumen samples. 

### 2.4. SARA Analysis

The four bitumen fractions (SARA) were identified as important properties of bitumen quality, and the SARA content differences influenced the bitumen recovery. In this study, the four fractions were carried out using ASTM D4124. The bitumen from single solvent extraction and composite solvent extraction were all analyzed by ASTM D4124. Asphaltene was the component that could dissolve into toluene, but could not dissolve into n-heptane [25]. First, 2.0 g bitumen was put into a 500 mL beaker, 100 mL n-heptane was added, and the bitumen was sonicated at 50 °C for 1 h. The mixture was centrifuged and the supernatant was transferred to a 500 mL flask. The insoluble solids were put into another flask. The process was repeated with 100 mL n-heptane until the supernatant was colorless. The insoluble solids were then dried and identified as asphaltene. The supernatant was placed under a rotary evaporator to obtain the mixtures (saturates, aromatics, and resins). The mixtures were eluted with different solvents following the literature [31].

### 2.5. Viscosity Measurement and Element Content Analysis

The viscosity of bitumen, by single and composite solvent extraction from crude bitumen samples, was measured by a viscometer (NDJ-5S, Shanghai Youyi Instrument Co. Ltd., Shanghai, China). The bitumen temperature was 50 °C. In the viscosity measurement, the relationship between bitumen viscosity and bitumen recovery was studied. The element content of bitumen from single solvent extraction and composite solvent extraction was analyzed by an elemental analyzer (vario micro cube, Elementar, Langenselbold, Germany), and the C, H, O, N, S element contents in different bitumen samples were measured.

## 3. Results and Discussion

### 3.1. Single Factor Experiment

The bitumen recovery increased quickly with increasing temperatures from 25 to 55 °C (black line). The increasing temperature caused the rate of bitumen dissolving into toluene to increase, which caused the bitumen recovery to increase. The bitumen recovery decreased when the temperature increased from 55 to 70 °C, due to the volatilization of toluene and the higher temperature influence on the bitumen recovery. The bitumen recoveries at 40, 55 °C were 96.5 wt%, 97.2 wt%. The bitumen recovery increase was low, so 40 °C was the optimal temperature.

The bitumen recovery increased from 88.2 to 96.5 wt% with the crude bitumen sample to solvent ratio increasing from 1:1 to 1:10 (red line). This is because the bitumen dissolution increases as the toluene content increases. The bitumen recovery increased 0.7 wt% when the toluene increased from 1:10 to 1:20. Thus, 1:10 was the optimal ratio.

The bitumen recovery initially increased after 100 rpm, and then reached a plateau as the stirring rate increased to 500 rpm (green line). This indicated that 100 rpm was the threshold stirring rate for the dissolution of bitumen in the organic solvent. The increasing stirring rate helped bitumen liberate from the minerals and dissolve into the toluene phase, though the bitumen recovery decreased 0.3 wt% as the stirring speed increased from 500 to 700 rpm. This was due to the increasing stirring rate influencing the SARA of bitumen, and then decreasing the bitumen recovery. Therefore, 500 rpm was the optimal stirring rate.

The bitumen recovery increased greatly when the stirring time increased from 20 to 60 min (yellow line) because the bitumen recovery process dissolved into toluene. The increased stirring time caused the bitumen dissolution to increase; however, when the stirring time increased from 60 min to 80 min, the bitumen recovery increased just 0.6 wt%. Therefore, 60 min was the optimal stirring time.

**Figure 3:**
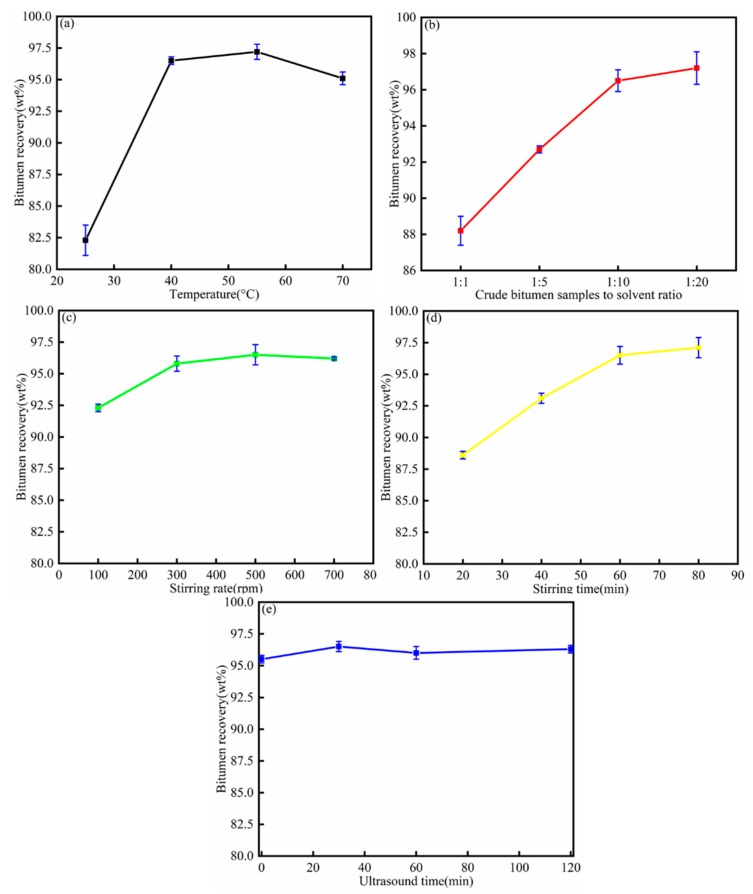
Influence of (**a**) temperature (black line), (**b**) crude bitumen sample to solvent ratio (red line), (**c**) stirring rate (green line), (**d**) stirring time (yellow line), and (**e**) ultrasound time (blue line) on the bitumen recovery from crude bitumen samples.

Figure 3e shows that bitumen recovery increased from 95.5 to 96.5 wt% when the ultrasound time increased from 0 to 30 min, because ultrasound promoted the bitumen dissolution process. The bitumen recovery decreased when the ultrasound time was higher than 30 min, which was due to the ultrasound cavitation effect influencing the mixture stability. Therefore, 30 min was the optimal ultrasound time.

In order to obtain a higher bitumen recovery by toluene, the optimal operation condition was as follows: the temperature was 40 °C, crude bitumen sample to solvent ratio was 1:10, stirring rate was 500 rpm, stirring time was 60 min, and ultrasound time was 30 min. The process parameters were used for the next single solvent extraction and composite solvent extraction.

### 3.2. Single Solvent Extraction Experiment

**Figure 4:**
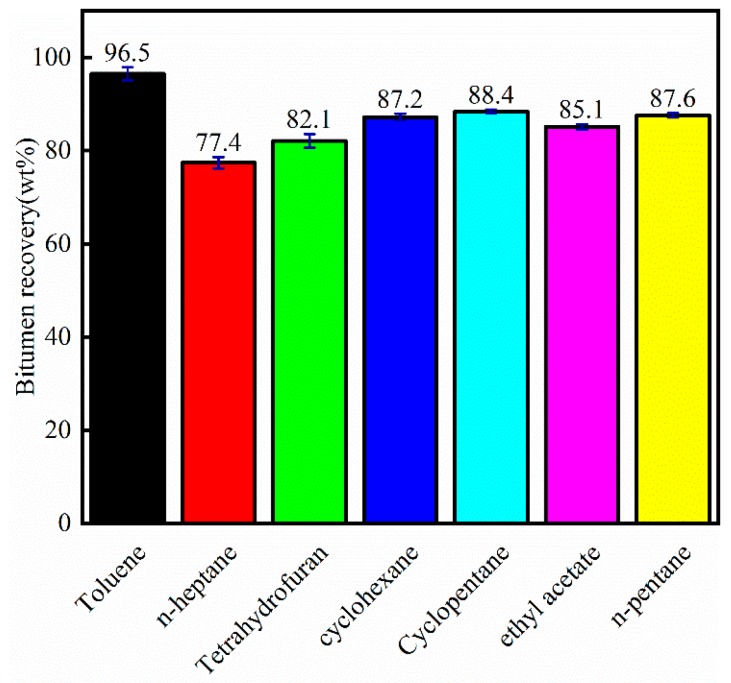
Final bitumen recovery by different solvents for crude bitumen samples.

The varying solvent extractions of bitumen were applied to study the effects of different solvents on bitumen recovery from crude bitumen samples, as shown in Figure 4. It was shown that the final bitumen recovery could reach at least 77.4% by n-heptane. Because n-heptane cannot dissolve asphaltene in bitumen, it could only dissolve the SAR (saturates, aromatics, and resins) components. Toluene showed the highest bitumen recovery (96.5 wt%) among these solvents, because it is an aromatic solvent that can dissolve bitumen components efficiently. Cyclohexane and cyclopentane obtained 87.2 and 88.4 wt% bitumen from crude bitumen samples, because cyclohexane and cyclopentane are cycloalkanes which are similar to the structure of aromatics and resins. When toluene or cycloalkanes were taken as solvents, most of the heavy components (e.g., resins and asphaltenes) could be dissolved into the solvent. However, for n-alkanes, bitumen heavy substances could not easily be extracted, so the bitumen recovery was low. Tetrahydrofuran, ethyl acetate, and n-pentane showed bitumen recovery among the toluene and n-heptane. Although toluene showed the highest bitumen recovery from crude bitumen samples, the toxicity of toluene limited the application. Therefore, finding an alternative solvent is a significant issue.

**Figure 5:**
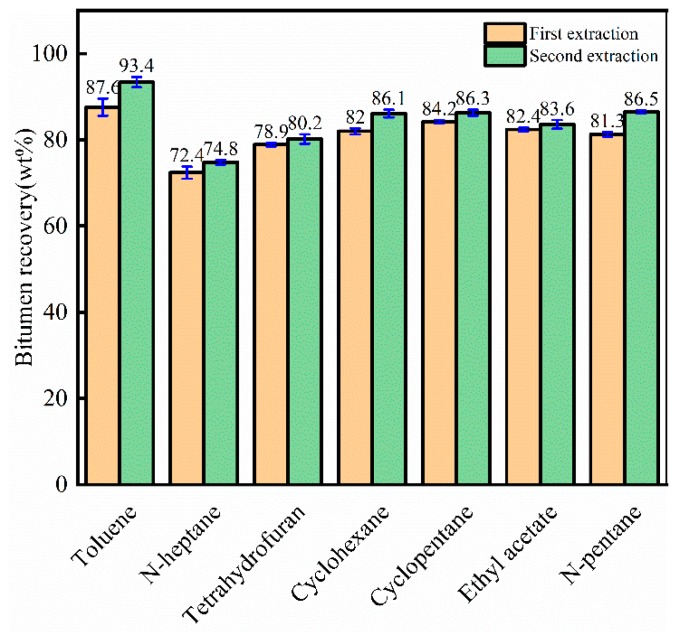
First and second bitumen recoveries by different solvents for crude bitumen samples.

Figure 5 indicates that increasing extraction times increase bitumen recovery. For different solvents, the influence of extraction time on bitumen recovery was varied. Bitumen recovery by toluene increased from 87.6 wt% (first extraction) to 93.4 wt% (second extraction), and the final bitumen recovery was 96.5 wt%, which is higher than other solvents. The final bitumen recovery by different solvents was in the order of toluene > cyclopentane > n-pentane > cyclohexane > ethyl acetate > tetrahydrofuran > n-heptane. The first bitumen recovery order was toluene > cyclopentane > ethyl acetate > cyclohexane > n-pentane > tetrahydrofuran > n-heptane. The second bitumen recovery order was toluene > n-pentane > cyclopentane > cyclohexane > ethyl acetate > tetrahydrofuran > n-heptane. For different extraction times, the bitumen recovery orders were different.

### 3.3. SARA Analysis of Bitumen from Single Solvent Extraction

**Figure 6:**
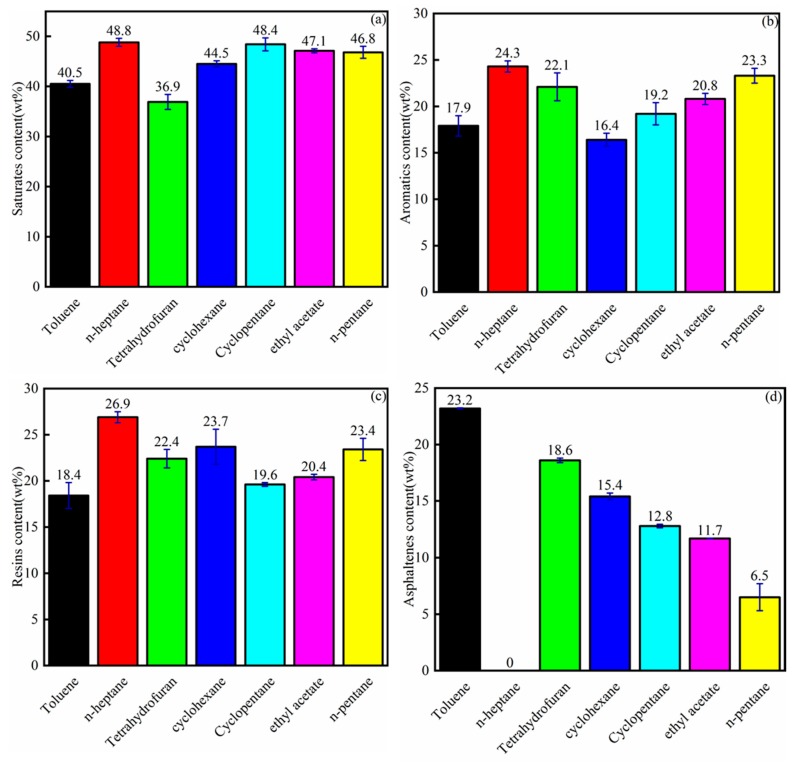
Saturates (**a**), aromatics (**b**), resins(**c**) and asphaltenes (**d**) contents of the bitumen from different single solvent extraction experiments.

Figure 6 shows the SARA contents of bitumen by different single solvent extractions. For saturates and aromatics, n-heptane and n-pentane showed high contents compared to other solvents, as shown in Figure 6a,b. The linear hydrocarbon dissolved the light components of bitumen. The bitumen by toluene extraction showed low saturate and aromatic contents, so the toluene dissolution of light components was low. The resin contents by n-heptane and n-pentane extraction were 26.9 and 23.4 wt%, which were higher than those obtained by toluene extraction (Figure 6c). These results indicate that linear hydrocarbons have a good solubilization with resins. The resin contents by toluene extraction showed the lowest bitumen recovery, which meant that toluene could not dissolve resin well. As shown in Figure 6d, the asphaltene content decreased from 23.2 to 0 wt% when the solvent was changed from toluene to n-heptane. Toluene was the aromatic solvent that showed the highest asphaltene content (23.2 wt%) among the solvents. The asphaltene content of the bitumen extracted by n-heptane was 0 wt%, because asphaltene is defined as the component that is insoluble in n-heptane but soluble in toluene. N-pentane obtained 6.5 wt% asphaltene. These results indicate that linear hydrocarbons do not have a good solubilization with asphaltene. The asphaltene contents obtained by tetrahydrofuran, cyclohexane, cyclopentane, and ethyl acetate extraction were 18.6, 15.4, 12.8, and 11.7 wt%, respectively. 

As shown in Figure 4 and Figure 6, toluene showed the highest bitumen recovery, but the bitumen (extracted by toluene) composition contained low saturate, aromatic, and resin contents. The asphaltene content obtained by toluene extraction was the highest. The dissolution of asphaltene by the solvent indicated the ability to obtain bitumen.

### 3.4. Composite Solvent Extraction

**Figure 7:**
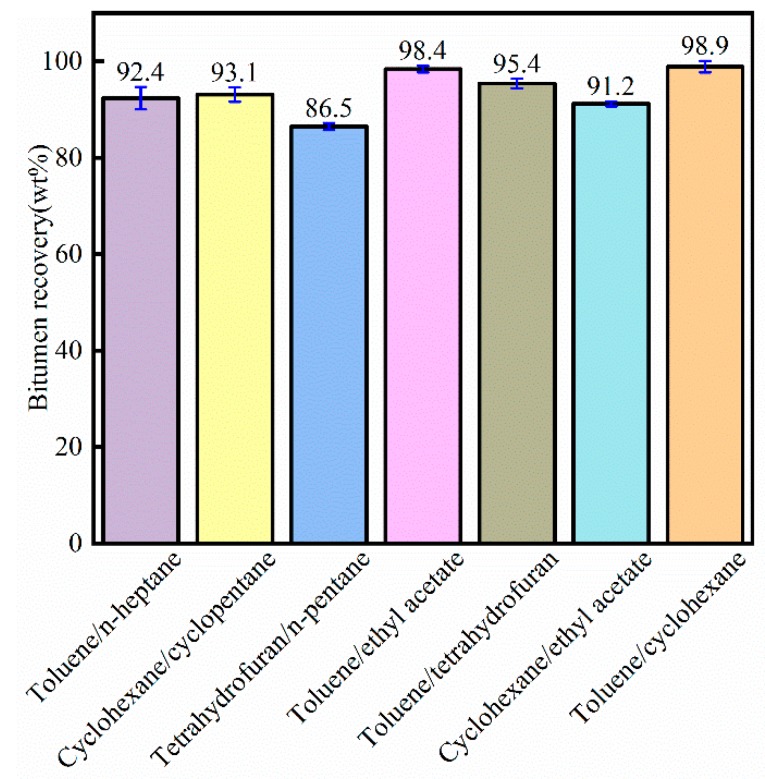
Bitumen recovery by composite solvents for crude bitumen samples.

As shown in Figure 7, the bitumen recovery increased significantly by composite solvent compared to the single solvent. In Figure 4, bitumen recovery was lower than 90 wt% (toluene exempt). In Figure 7, bitumen recovery was higher than 90 wt% (tetrahydrofuran/n-pentane exempt). The highest bitumen recovery for composite solvent was 98.9 wt% (toluene/cyclohexane) because toluene could dissolve the asphaltene component and cyclohexane could dissolve SAR components. Therefore, the toluene/cyclohexane composite solvent showed the highest bitumen recovery. 

The bitumen recovery extracted by composite solvents including toluene, namely toluene/n-heptane, toluene/ethyl acetate, toluene/tetrahydrofuran, and toluene/cyclohexane, was higher than the bitumen extracted by single solvent extraction.

### 3.5. SARA Analysis of Bitumen from Composite Solvent Extraction

**Figure 8:**
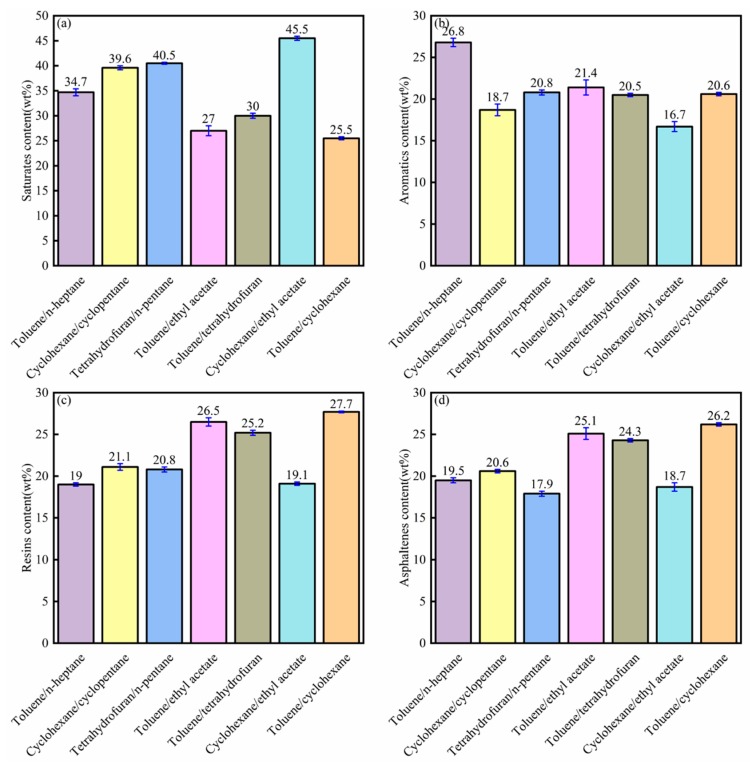
Saturate (**a**), aromatic (**b**), resin, and (**c**) asphaltene (**d**) contents of the bitumen from different composite solvent extraction experiments.

Figure 8 shows the relative contents of SARA fractions of bitumen from different composite solvent extractions. As shown in Figure 6d and Figure 8d, the asphaltene content increased significantly from single solvent extraction to composite solvent extraction. 

Among the composite solvents, toluene/cyclohexane showed the highest asphaltene content, while tetrahydrofuran/n-pentane showed the lowest asphaltene content. During the extraction process, the asphaltene content was closely related to the bitumen recovery. The higher the asphaltene content, the higher the bitumen recovery.

### 3.6. Viscosity Analysis

**Figure 9:**
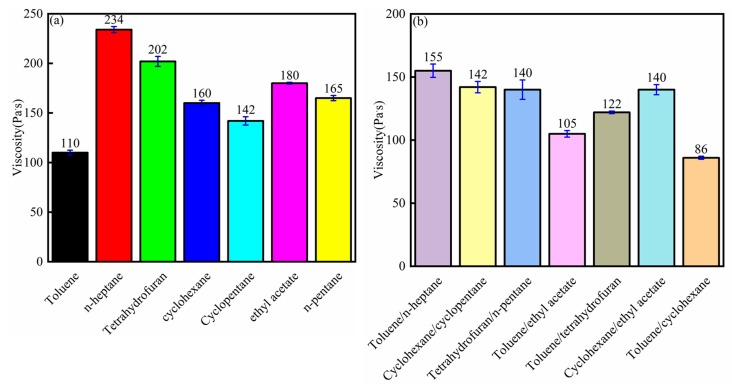
Viscosity analysis of bitumen from (**a**) single solvent extraction; (**b**) composite solvent extraction.

The bitumen samples that were extracted from different solvents showed different viscosities. The low bitumen viscosity helped liberate bitumen from minerals. Figure 9a indicates that the bitumen extracted from toluene showed the lowest viscosity (110 Pa·s), and the bitumen extracted from n-heptane showed the highest viscosity (234 Pa·s). Toluene dissolved the heaviest component (asphaltenes), so the viscosity decreased. The cyclic hydrocarbons (cyclohexane and cyclopentane) showed low viscosities (160 and 142 Pa·s). The bitumen viscosity decreased significantly according to the composite solvent, as shown in Figure 9b. The lower the bitumen viscosity, the higher the bitumen recovery. Bitumen is a non-Newtonian fluid, and the relationship between the shearing force and shear rate was not linear. Bitumen mixtures showed linear viscoelastic properties under low temperatures and small deformations, but showed nonlinear viscoelastic properties under high temperatures and large deformations. Many researchers have focused on the mechanical properties of bitumen [29,30]. Bazzaz put forward a straightforward procedure to characterize the nonlinear viscoelastic response of asphalt concrete at high temperatures [29]. Darabi put forward a new model that could accurately describe the asphalt material behavior under different loading paths, and this model can help in experiment design [30]. 

### 3.7. The Element Content Analysis of Bitumen

As shown in Table 3, the bitumen by toluene extraction showed the highest C/H ratio, while bitumen by the n-heptane extraction showed the lowest C/H ratio. This was due to the fact that toluene could dissolve heavy components (asphaltene), and the C/H in asphaltene is higher than the ratios in other components; meanwhile, n-heptane could only dissolve the SAR components. The C/H ratio for different solvents was different, which influenced bitumen recovery. The C content extracted by composite solvent was higher than that extracted by the single solvent, as shown in Table 4. The highest C/H ratio was achieved by toluene/cyclohexane extraction. The higher the C/H ratio, the higher the bitumen recovery.

## 4. Conclusions

This research put forward a new method to solve the heterogeneous formation of bitumen occurring during the oil-gas drilling process. In order to alleviate the deterioration of drilling fluid performances caused by the formation of bitumen and to eliminate the negative influence of bitumen adhesion in time, we studied the solvent extraction process from crude bitumen samples taken from Halfaya oilfield (Iraq). The relation between the solvent and bitumen was analyzed. It was concluded that:

(1) The optimal operation condition for bitumen recovery by toluene from crude bitumen samples was, as follows: the temperature was 40 °C, crude bitumen sample to solvent ratio was 1:10, stirring rate was 500 rpm, stirring time was 60 min, ultrasound time was 30 min.
(2) The bitumen recovery increased significantly using the composite solvent compared to the single solvent. The highest bitumen recovery from crude bitumen samples was 98.9 wt%. SARA analysis indicated that the asphaltene content increased significantly from the single solvent to composite solvent. Toluene and cycloalkane showed the highest asphaltene content, while the n-alkanes showed the lowest asphaltene content. The composite solvent obtained the highest asphaltene content and bitumen recovery.
(3) The bitumen samples extracted from different solvents showed different viscosities. The bitumen viscosity influenced the bitumen recovery, and the lower the bitumen viscosity, the higher the bitumen recovery. The C/H ratio of the bitumen followed this rule.

## Figures and Tables

**Table 1:** The list of experimental conditions for the single factor experiment.

Variable Factors	1	2	3	4	Other Factors
Temperature (°C)	25	40	55	70	Condition a
Oil sands to solvent ratio	1:1	1:5	1:10	1:20	Condition b
Stirring rate (rpm)	100	300	500	700	Condition c
Stirring time (min)	20	40	60	80	Condition d
Ultrasound time (min)	0	30	60	120	Condition e

**Table 2:** The detailed description for conditions a, b, c, d, e in Table 1.

Condition	Temperature (°C)	Oil Sands to Solvent Ratio	Stirring Rate (rpm)	Stirring Time (min)	Ultrasound Time (min)
a	Variable	1:10	500	60	30
b	40	Variable	500	60	30
c	40	1:10	Variable	60	30
d	40	1:10	500	Variable	30
e	40	1:10	500	60	Variable

**Table 3:** The elemental analysis of bitumen (wt%) from single solvent extraction.

Bitumen from Single Solvent Extraction	C	H	O	N	S
toluene	83.358	9.650	0.072	0.490	6.430
n-heptane	79.652	12.152	2.766	0.347	5.083
tetrahydrofuran	81.642	11.048	2.623	0.322	4.365
cyclohexane	82.658	10.346	0.028	0.432	6.536
cyclopentane	82.356	10.586	1.481	0.440	5.137
ethyl acetate	81.568	11.036	2.874	0.354	4.168
n-pentane	82.265	10.952	1.057	0.406	5.320

**Table 4:** The elemental analysis of bitumen (wt%) from composite solvent extraction.

Bitumen from Composite Solvent Extraction	C	H	O	N	S
toluene/n-heptane	83.532	10.564	0.442	0.379	5.083
cyclohexane/cyclopentane	83.048	10.298	1.509	0.265	4.889
tetrahydrofuran/n-pentane	82.653	11.892	0.662	0.336	4.458
toluene/ethyl acetate	84.685	8.780	0.455	0.405	5.675
toluene/tetrahydrofuran	84.068	9.365	0.094	0.376	6.097
cyclohexane/ethyl acetate	82.964	11.068	0.021	0.983	4.964
toluene/cyclohexane	85.026	8.460	0.803	0.344	5.367
